# Der Wearable Kardioverter-Defibrillator als Diagnostikum: Fallbeispiel aus dem telemedizinischen Alltag

**DOI:** 10.1007/s00399-021-00769-0

**Published:** 2021-05-12

**Authors:** Tobyson Pulickal, Thomas M. Helms, Christian A. Perings

**Affiliations:** 1 Katholisches Klinikum Lünen / Werne St.-Marien-Hospital Lünen, Medizinische Klinik 1, Lünen, Deutschland; 2 Deutsche Stiftung für chronisch Kranke, Fürth, Deutschland; 3 Peri Cor Arbeitsgruppe Kardiologie/Ass. UCSF, Hamburg, Deutschland

**Keywords:** Tragbare Defibrillator-Weste, Telemedizin, Herzinsuffizienz, Pulmonalvenenisolation, Prognoseverbesserung, Wearable defibrillator vest, Telemedicine, Heart failure, Pulmonary vein isolation, Prognosis improvement

## Abstract

Eine telemedizinische Versorgung kann neue Einsatzmöglichkeiten bereits etablierter Therapeutika wie einer tragbaren Defibrillator-Weste (WCD) ermöglichen und über ein verbessertes Management von hierüber abgeleiteten Vitaldaten die Versorgungsqualität von chronisch kranken Patienten mit Herzinsuffizienz (HI) erhöhen. Im aktuellen Fallbericht wird der klinische Verlauf eines 71 Jahre alten Patienten beschrieben, der nach einer akuten kardialen Dekompensation und neudiagnostizierter hochgradiger Einschränkung der kardialen Pumpfunktion im Rahmen einer ischämischen Kardiomyopathie leitliniengerecht mit einer WCD bis zur endgültigen Entscheidung über eine ICD-Implantation versorgt wurde. Die durch die WCD gesammelten Vitalwerte wurden über ein Telemedizinzentrum (TMZ) strukturiert ausgewertet und hierbei ein Rezidiv des vorbekannten paroxysmalen Vorhofflimmerns (VHF) entdeckt, was vor einer möglichen Dekompensation durch Anpassung der Medikation und frühzeitiger Initiierung einer Pulmonalvenenisolation (PVI) therapiert werden konnte. Dieser Fall zeigt exemplarisch die Sinnhaftigkeit strukturierter telemedizinischer Intervention auf, die es ermöglicht, etablierte Konzepte der Patientenversorgung sinnvoll zu ergänzen, bestehende Konzepte zu optimieren und die Patientenversorgung signifikant zu verbessern.

## Abstract (en)

Telemedical care can point out new applications of already established therapeutics such as a wearable cardioverter–defibrillator (WCD) and improve the quality of care for chronically ill patients with heart failure through improved management of data derived from this device. The current case report describes the clinical course of a 71-year-old man, who was treated with a WCD in accordance with the current guidelines until a final decision was made about ICD implantation, after acute cardiac decompensation and lately diagnosed severe decrease of left ventricular function in the context of ischaemic cardiomyopathy. The data collected by the WCD were evaluated in a structured way via a telemedicine centre (TMC) and recurrence of the previously known paroxysmal atrial fibrillation (AF) was discovered. This made it possible to treat the paroxysmal AF, before cardiac decompensation happened again by adjusting the medication and early initiation of pulmonary vein isolation (PVI). This case exemplifies the usefulness of structured telemedical intervention, which makes it possible to meaningfully supplement established concepts of patient care, improve existing concepts and significantly improve patient care.

Seit längerer Zeit steht mit der tragbaren Defibrillator-Weste (WCD) ein Medizinprodukt zur Verfügung, welche Patienten mit einem passager erhöhtem Risiko für einen plötzlichen Herztod wirksam vor potenziell letalen Rhythmusereignissen schützen kann [1–3]. Auch wenn zuletzt die therapeutische Wirkung der WCD nicht sicher belegt werden konnte [4, 5] und weitere randomisierte Studien gefordert werden, so kann mit zusätzlichen Funktionalitäten hinsichtlich Vitaldatenüberwachung geeigneter Patienten eine Erweiterung der WCD-Indikation weg von einem reinen Therapeutikum hin zu einem Therapeutikum mit diagnostischen Eigenschaften erfolgen. Aufgrund der vorhandenen Fähigkeiten der WCD der Fa. Zoll bietet sich in diesem Fall eine Verbindung mit dem sich dynamisch entwickelnden Feld der Telemedizin an. Hierfür verfügt die WCD bereits über eine etablierte Plattform mit dem Namen „LifeVest Network“, auf welcher sich jenseits der bekannten EKG-Befunde, weitere Vitaldaten darstellen lassen, die über die WCD abgeleitet werden.

Insbesondere bei Patienten mit einer Herzinsuffizienz (HI), bei welchen die WCD vorrangig eingesetzt wird, mehrt sich die Evidenz, dass der strukturierte Einsatz von Telemedizin zu einer Verbesserung sowohl patientenorientierter Quality-of-life-Parameter (QoL) als auch zu einer Reduktion von Krankenhausaufenthalten und Mortalität in dieser Patientengruppe kommen kann [6, 7].

In dem vorliegenden Fall soll eine solche telemedizinisch-assistierte Intervention mit Hilfe einer WCD der Fa. Zoll in Verbindung mit einem TMZ vorgestellt werden, bei welchem die zusätzlichen Monitoring-Fähigkeiten der WCD einerseits und andererseits die telemedizinische Intervention jenseits der bloßen Datenakquise dargestellt werden.

## Patientenvorstellung

Der betrachtete Patient ist zum Zeitpunkt der Intervention in unserem Krankenhaus (Mai bis Juli 2020) 71 Jahre alt, männlichen Geschlechts und nicht mehr berufstätig.

Vorbekannt war eine dialysepflichtige Niereninsuffizienz seit > 1 Jahr auf dem Boden eines kardiorenalen Syndroms, eine schwere koronare Dreigefäßerkrankung, eine sauerstoffpflichtige chronisch-obstruktive Lungenerkrankung (COPD) und ein paroxysmales Vorhofflimmern (VHF), welches mittels Marcumar antikoaguliert wurde.

**Figure 1:**
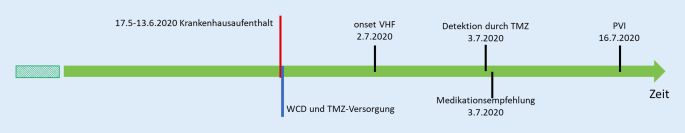

Zum Aufnahmezeitpunkt am 17.05.2020 zeigte sich der Patient in einem guten Ernährungs- bei gleichzeitig reduziertem Allgemeinzustand (Abb. 1). Es fanden sich bei fehlenden, pathologischen Herzgeräuschen und rhythmischer Herzaktion, klingende Rasselgeräusche im linken Lungenflügel im Bereich des Mittelfelds. Zudem fand sich eine Klopfschallabschwächung bds. basal. Im EKG konnte ein normofrequenter Sinusrhythmus detektiert werden.

Die initiale Vorstellung erfolgte aufgrund einer progredienten Luftnotsymptomatik. Nachdem mittels PCR-Abstrich eine SARS-CoV-2-Infektion ausgeschlossen werden konnte, zeigte sich radiomorphologisch eine flächenhafte Infiltration im Sinne einer Pneumonie links pulmonal sowie bilaterale Pleuraergüsse.

Nach Behandlung der Pneumonie wurde eine Echokardiographie durchgeführt. Obwohl initial ein normofrequenter Sinusrhythmus zu dokumentieren war, war während der Untersuchung ein normofrequentes Vorhofflimmern (VHF) zu sehen. Hierbei zeigte sich eine hochgradig reduzierte Pumpfunktion mit einer Ejektionsfraktion (EF) von 32 % (biplan). Da sich diese im Vergleich zu den Vorbefunden verschlechtert hatte, wurde eine diagnostische Koronarangiographie durchgeführt, in welcher sich nun eine ca. 50 %ige Stenose des linken Hauptstammes zeigte, die in der FFR-Messung eine hämodynamische Relevanz aufwies.

Nach Intervention (s. unten) zeigte sich unter Sinusrhythmus eine persistierende hochgradig eingeschränkte Pumpfunktion. Initial wurde eine intravenöse antibiotische Therapie mit einem 3. Generation-Cephalosporin (Ceftriaxon) initiiert und die Pleuraergüsse mittels Pleurapunktion entlastet.

Nach abgeheilter Pneumonie und nach der diagnostischen Koronarangiographie wurde eine Vorstellung in einer gemeinsamen Kardiochirurgischen Konferenz aus Kardiologen und Kardiochirurgen zur Planung des weiteren Prozederes veranlasst. Hierbei war aufgrund des erhöhten Operationsrisikos ein interventioneller Therapieversuch der Hauptstammstenose beschlossen worden. Unter Einsatz einer Mikroaxialpumpe zur Unterstützung der Pumpleistung wurde eine erfolgreiche Hauptstammintervention durchgeführt.

Hiernach wurde der Patient überwacht, wobei sich wieder ein stabiler Sinusrhythmus zeigte. Da die hochgradig eingeschränkte Pumpfunktion persistierte, wurde eine Versorgung mit einer WCD der Fa. Zoll initiiert und die Herzinsuffizienzmedikation den aktuellen Leitlinien entsprechend optimiert [8].

Gleichzeitig erfolgte die Registrierung des Patienten im „Westdeutschen Zentrum für angewandte Telemedizin (WZAT)“ als DGK-zertifiziertes TMZ, welches sich fortan im Rahmen einer Kooperation mit der Fa. Zoll um das ambulante Management der durch die WCD ermittelten Vitaldaten und das entsprechende ambulante Patientenmanagement kümmerte. Am 13.06.2020 erfolgte schließlich die Entlassung in die Häuslichkeit.

Im Rahmen von täglichen digitalen Visiten wurden die übermittelten Vitalwerte der WCD durch die Medizinischen Fachangestellten (MFA) und die im TMZ tätigen Ärzte vidiert. Zudem war der Patient angehalten, selbstständig drei Mal täglich ein EKG mit Hilfe der WCD aufzuzeichnen.

Insbesondere zu Beginn der WCD-Versorgung benötigte der Patient häufiger telefonische Unterstützung im Umgang mit der WCD, da ihm insbesondere das Anlegen schwerfiel und somit häufige akustische Alarme provoziert wurden.

Durch das Monitoring der Tragezeit und der daraus resultierenden telefonischen Kontaktaufnahmen konnte trotz der beschriebenen Probleme eine hohe Compliance mit einer durchschnittlichen Tragezeit von über 22 h/Tag erreicht werden.

**Figure 2:**
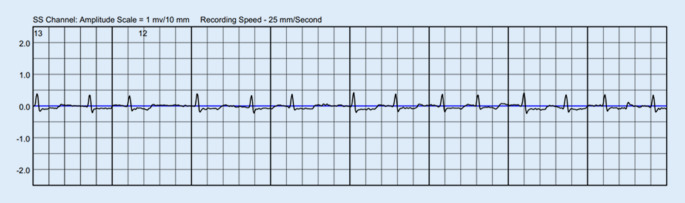

Daneben konnte in den übermittelten EKGs ein Wiederauftreten von VHF dokumentiert werden (Abb. 2).

**Figure 3:**
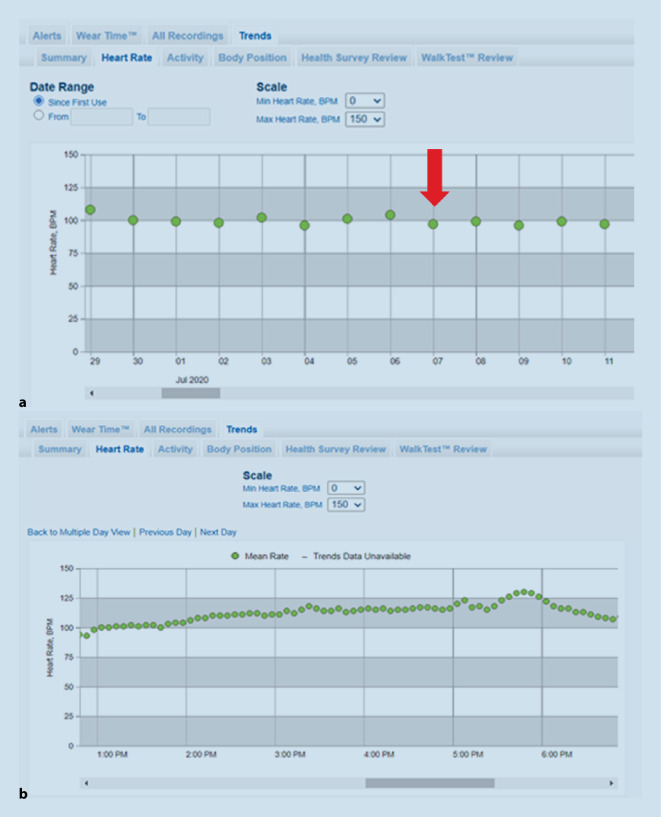

Da darüber hinaus die durchschnittliche Herzfrequenz > 100/min in 24 h und sich eine in der Detailansicht akzelerierende Tendenz zeigte (Abb. 3), wurde dem Patienten zunächst eine Erhöhung der bereits bestehenden Betablockertherapie empfohlen.

Zudem wurde der Patient wieder an unsere Klinik zurück überwiesen mit der Bitte einer Reevaluation der Frequenz- bzw. des Versuchs einer Rhythmuskontrolle. Aufgrund der HI wurde unter prognostischen Gesichtspunkten eine PVI terminiert und erfolgreich durchgeführt.

## Follow-up/Outcome

Da der Patient telemedizinisch mitbetreut wurde, konnte zunächst eine hohe Compliance erreicht werden. Durch die erfolgreiche PVI und die zuvor rasch erfolgte Medikationsanpassung durch die Telemediziner konnte eine erneute kardiale Dekompensation und vorzeitige Hospitalisierung des Patienten verhindert werden. Eine Verbesserung der Pumpleistung stellte sich leider nicht ein, so dass letztlich ein Defibrillator implantiert werden musste.

Während der gesamten telemedizinischen Betreuungsphase konnte durch das stetige und strukturierte Überwachen und Verarbeiten der eingegangenen Vitalwerte mit entsprechender Reaktion eine erneute kardiale Dekompensation verhindert werden.

## Diskussion

Patienten mit einer HI sind komplex in der Versorgung. Neben der Herausforderung im Hinblick auf die optimale Dosierung der durch die Leitlinien empfohlenen Pharmakotherapie [8] ist ein in Metaanalysen nachgewiesener positiver Effekt im Hinblick auf Interventionen zu erfassen, welche mit dem Ziel einer adäquaten Adhärenz hinsichtlich Medikamenteneinnahme und Dossierung durchgeführt worden sind [9]. Daher ist der Ansatz einer fortwährenden Überwachung als sinnvolles Ziel anzusehen.

Das wie in dem vorgestellten Fallbeispiel vorkommende VHF kann im Rahmen einer bereits bestehenden HI die Prognose verschlechtern [10], wobei mittlerweile wissenschaftliche Evidenz aus der CASTLE-AF-Studie existiert, dass eine PVI bei geeigneten Patienten einen Vorteil hinsichtlich Hospitalisation und Verschlechterung der HI erbringen kann [11]. Daneben ist eine Kontrolle der Herzfrequenz im Rahmen der HI sinnvoll, wobei die optimale Herzfrequenz für Patienten mit HI und VHF unklar ist und in den aktuellen Leitlinien zwischen 70 und 100/min angegeben wird, wohingegen bei Patienten mit HI und Sinusrhythmus eine Frequenz von < 70/min als Ziel erachtet wird [8, 12]. Dies illustriert einmal mehr die Notwendigkeit einer individualisierten Diagnostik und Therapie im Kollektiv der Patienten mit HI.

Die Verwendung einer WCD mit erweiterten diagnostischen Funktionen als Mittel zur Überwachung eben dieses Kollektivs ist bereits Gegenstand wissenschaftlicher Untersuchungen und hat das Potenzial, den Anteil nicht adäquat therapierter Patienten in einem mit der WCD versorgten Kollektiv aufzuzeigen [13], so dass diese einer Anpassung ihrer Therapie zugeführt werden können. Neben der reinen Rhythmusüberwachung sind Beschleunigungssensoren implementiert, welche die Erfassung verschiedener weiterer Parameter wie „Gehstrecke“ oder „6-Minuten-Gehtest“ ermöglichen, die sich für die Verlaufsbeobachtung von HI-Patienten eigenen. Diese Verlaufsparameter müssen nicht mehr im Krankenhaus, sondern können in der eigenen Lebenswirklichkeit durchgeführt werden [14, 15].

Daneben findet sich auch neben temporären Devices wie der WCD ebenso für bereits im Rahmen der leitliniengerechten Versorgung implantierten dauerhaften Devices, welche telemedizinfähig sind, ein positiver Effekt auf das Outcome [16], wenn diese mit einer telemedizinischen Versorgung kombiniert worden sind.

Die in dem vorgestellten Fallbeispiel erhobenen Rhythmusdaten über die WCD sind in der klinischen Praxis bereits vorhanden, doch mangelt es oft an Strukturen, die eine geordnete Interpretation und Reaktion auf die empfangenen Daten zeigen. Zudem lebt ein System wie die WCD von einer ausreichenden Compliance, da es sonst nicht im Sinne eines Therapeutikums wirken kann [17]. Beide Anforderungen konnten in unserem Fallbeispiel durch die Mitversorgung bzw. die Intervention des TMZ erreicht werden. Dies zeigt, dass die telemedizinische Versorgung einer gewissen Struktur und Qualität bedarf, um entsprechende Ergebnisse zu erzielen [18, 19]. Dass Telemedizin jenseits des auswertenden einen manifesten therapeutischen Einfluss auf Mortalität und Morbidität haben kann, zeigte sich zuletzt in einer Nachbeobachtung des TIM-HF2-Kollektivs nach Beendigung der telemedizinischen Versorgung. Hierbei war nämlich der positive Effekt der telemedizinischen Versorgung hinsichtlich Mortalität und Morbidität verschwunden [20].

Aufgrund der technischen Beschränkung der WCD und der Unfähigkeit, Rhythmusereignisse unterhalb der programmierten VT-Schwelle zu detektieren, wären die in dem Fallbeispiel erhobenen Daten eines grenzwertig schnellen VHF ohne eine aktive Überwachung der eingehenden Vitalwerte und ohne ein regelmäßig durch den Patienten angefertigtes EKG mit entsprechender Interpretation durch ein TMZ nicht auffällig geworden und hätten keine Anpassung der Therapie ausgelöst.

Ein System wie die WCD könnte in Zukunft nicht nur anhand der adäquaten Schocks beurteilt werden, sondern in Kombination mit strukturierter Telemedizin auch nach deren Fähigkeit, sensorische Daten zu liefern, durch die eine bessere Versorgung immer komplexer werdender Patienten möglich ist.

Daher ist eine regelhafte Kombination eines solchen Systems mit einer strukturierten telemedizinischen Mitversorgung, wie in dem dargestellten Fallbeispiel, zielführend in Bezug auf eine verbesserte Patientenversorgung.
